# Bridging the Gap: Resident Doctor Patient Safety Representatives in a Mental Health Trust

**DOI:** 10.1192/bjo.2026.11440

**Published:** 2026-06-30

**Authors:** Idura Hisham, Sanchari Mukhopadhyay, Rebecca Garvey, Lena Rane

**Affiliations:** South London and Maudsley NHS Foundation Trust, London, United Kingdom

## Abstract

**Aims:**

To address (the outcome of) a survey (on patient safety) indicating resident doctors atthe Trust did not feel able to raise patient safety concerns, or that if they did these concerns would not be addressed.To increase resident doctor satisfaction by 20% in two years and improve two-way communication with Trust patient safety infrastructure.

**Methods:**

This project utilised a sequential Quality Improvement (QI) methodology. The steps undertaken:

1. Baseline Assessment:Collated from the NHS national staff survey, Trust-wide DATIX reporting trends and specific survey for resident doctors to identify specific cultural and procedural barriers to safety reporting.

2. Governance and Advocacy:Representatives attended the monthly Trust Patient Safety Committee to represent frontline concerns. They acted as accessible points of contact for trainees, ensured “closed-loop” feedback on raised issues, and provided peer-led encouragement for formal DATIX reporting when indicated.

3. Closed-Loop Communication:A regular “Patient Safety Bulletin” was implemented to summarise key learnings, systemic issues, and specific resolutions using a transparent “You Said, We Did” framework.

4. Leadership Development:A programmewas launched allowing higher resident doctors to join internal Service Quality Reviews (SQR) as team members, to improve their experience of involvement in clinical governance.

5. Sustainability:To ensure project longevity, the representatives created a formal job description and offered the opportunity for resident doctors to undertake the role each year.

6. Evaluation:A repeat survey was distributed to resident doctors to measure the impact of the intervention against baseline metrics.

**Results:**

Resident doctors’ satisfaction with the reporting process rose from 13.5% (n=5/37) in 2025 to 65% (n=13/20) and confidence in effective action increased from 13.5% (n=5/37) to 60% (n=12/20) in 2026. 95% of resident doctors undertaking the survey found the trainee representative role and their actions helpful. Higher trainees provided positive feedback (n=7/7) on SQR involvement experience.

Other outcomes include resolution of a cross-site emergency protocol discrepancy, optimisation and promotion of ‘Consultant connect’ to enhance access to specialist physical health advice, and resident doctor representation at the Physical Health and Resus Committee.

**Conclusion:**

Peer-led representation provides a structured mechanism to reconcile the gap between safety awareness and clinical action. By formalising the feedback loop through transparent communication and creation of opportunities for involvement, this model addresses the perceived futility of reporting and establishes a sustainable pipeline for medical leadership in (clinical) safety governance. This approach offers a reproducible framework for enhancing safety culture and trainee engagement across mental health services.

